# Two cancer stem cell‐targeted therapies in clinical trials as viewed from the standpoint of the cancer stem cell model

**DOI:** 10.1002/sctm.19-0424

**Published:** 2020-04-12

**Authors:** Ingrid W. Caras

**Affiliations:** ^1^ California Institute for Regenerative Medicine Oakland California USA

**Keywords:** cancer stem cells, clinical trials, drug target, leukemia, immunotherapy

## Abstract

A key implication of the cancer stem cell model is that for a cancer therapy to be curative, it is imperative to eliminate the cancer stem cells (CSCs) that drive tumor progression. The California Institute for Regenerative Medicine is supporting two novel approaches that target CSCs, one an antibody‐mediated immunotherapy targeting CD47 and the other an antibody targeting ROR1. This article summarizes the evidence that CSCs are targeted and discusses the results of early clinical trials within the context of the CSC model.

## Graphical abstract

The Cancer Stem Cell (CSC) model asserts that tumors contain a subpopulation of CSC that drive tumor progression, positing that standard therapies are rarely curative because they spare the CSC, resulting in relapse after remission. Therapies that target primarily CSC are predicted to result in stable disease (SD) when used alone but are anticipated to induce durable responses when combined with standard therapies.

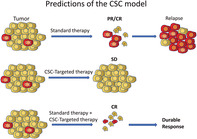

**Significance statement**

The premise and predictions of the cancer stem cell model of cancer are being tested in the clinic as cancer stem cell‐targeted therapies enter clinical trials. This article describes two such approaches and discusses whether the initial clinical results are consistent with predictions of the model. Validation of the cancer stem cell model in humans has implications for the design of curative treatments for many human cancers.

## 1 INTRODUCTION

The California Institute for Regenerative Medicine (CIRM), California's stem cell funding agency, selects highly meritorious projects using a rigorous peer review process and then partners with grantees to develop novel stem cell‐based treatments, including therapies aimed at eradicating cancer stem cells (CSCs). This article highlights two such approaches that are now in clinical trials.

## 2 CANCER STEM CELL MODEL

The CSC model asserts that tumors are comprised of heterogeneous hierarchies of cancer cells, not all of which are capable of sustaining tumor growth or initiating new tumors. Rather, there exists within most tumors a unique subset of cells, termed CSCs, that have stem cell‐like properties such as relative quiescence as well as ability to self‐renew and differentiate. It is these cells that drive both tumor progression and metastasis.^1^

Evidence for the existence of CSCs was first provided in acute myelogenous leukemia (AML)^2^ and has since been demonstrated in many other cancers.^1^ CSCs from human tumors have largely been identified based on their ability to propagate tumors in murine xenograft models. Using such models, AML CSCs, also known as leukemic stem cells (LSC), have been defined based on their ability to (a) engraft and form tumors after primary transplantation into immunodeficient mice, (b) propagate and form tumors in serial transplants, and (c) recapitulate the heterogeneity of the tumors from which they were derived including giving rise to non‐LSC progeny that do not engraft.^3^

Phenotypic as well as functional overlap between normal tissue stem cells and CSCs has been described in a number of cancers. AML LSC exhibit cell surface marker expression patterns characteristic of normal hematopoietic stem or progenitor cells.^3^ A gene expression signature specific for normal adult intestinal stem cells identified a population of colorectal CSCs that, as with AML LSC, robustly propagated tumors in immunodeficient mice and recapitulated the organization of the tumor of origin.^4^ Expression of a stem cell‐like signature in breast and colorectal tumors is predictive of more aggressive disease,^4^, ^5^ a finding consistent with the CSC model. Furthermore, a recent study integrating both gene expression and epigenetic features of multiple human cancers identified a “stemness index” to quantify stemness and found it to be most prominent in metastatic tumors.^6^

The CSC model can explain why most cancers recur after remissions induced by standard anticancer therapies. Stem cell‐like characteristics, such as relative quiescence as well as elevated levels of multidrug resistance transporters and DNA damage repair enzymes, enable CSCs to withstand chemotherapy or radiation and subsequently repopulate tumors and drive relapse.^7^ Consistent with this notion, LSC were shown to persist in bone marrow of AML patients following chemotherapy, even in patients in morphologic remission^8^ and there is evidence that the LSC population expands after relapse as predicted by the CSC model.^9^

In the case of targeted therapies, expression of the target on more differentiated progeny cells within the tumor but not on CSCs would also spare CSCs and allow for relapse.

A clear corollary of the CSC model is that a cancer therapy will never be curative unless the CSC population is eliminated. Many efforts are therefore underway to develop CSC‐targeted approaches.^10^ In particular, several CSC signaling pathways and regulators of stemness have been identified and agents targeting those pathways are currently undergoing clinical evaluation (reviewed in Reference 11). This article discusses two novel approaches being advanced by CIRM and its grantees. The evidence for CSC targeting is summarized below and emerging clinical results are examined in light of the CSC model.

## 3 CD47 AS A CSC TARGET

CD47 is expressed widely on both cancer and normal cells throughout the body.^12^, ^13^ It was initially identified as a potential CSC target by the observations that CD47 expression is elevated on AML LSC compared with normal bone marrow stem cells and high CD47 expression at diagnosis predicts worse overall survival (OS) in AML patients.^14^ The importance of CD47 for LSC‐driven tumor formation was demonstrated in a series of xenograft transplantation experiments in which precoating of human AML tumor cells with an anti‐CD47 antibody prevented leukemic engraftment in immunodeficient mice.^14^ Furthermore, when mice carrying established human AML tumors were treated with the anti‐CD47 antibody, there was almost complete elimination of circulating human AML LSC as well as a significant decrease in LSC remaining in the bone marrow. Secondary transplants from anti‐CD47‐treated mice resulted in no leukemic engraftment, further indicating that AML LSC had been eliminated.^14^ Parallel experiments using cells from human acute lymphoblastic leukemia (ALL) patients showed similar inhibition of leukemia formation by the anti‐CD47 antibody.^15^ Collectively, these experiments verified CD47 as an LSC target and provided preclinical proof‐of‐concept for CD47 blockade as a strategy to target LSC.

This strategy has been extended to other cancers. Overexpression of CD47 is correlated with poor prognosis in non‐Hodgkin's lymphoma (NHL), ovarian cancer, gastric cancer, and lung cancer. In xenograft models of multiple patient‐derived solid tumors, treatment with an anti‐CD47 antibody inhibited tumor growth and prevented metastasis, consistent with an effect on CSCs.^16^

## 4 CD47 BLOCKADE MECHANISM OF ACTION

CD47 functions as a ligand for signal regulatory protein‐α (SIRPα) on phagocytic macrophages, transmitting a “don't eat me” signal that inhibits phagocytosis.^12^, ^13^ Macrophage phagocytosis is determined by the balance between various prophagocytic and antiphagocytic signals. Overexpression of CD47 increases the net antiphagocytic signal and appears to be a general mechanism used by cancer cells to evade phagocytosis.^16^ Blocking CD47 presumably tips the balance in favor of phagocytosis and as predicted, disruption of the CD47‐SIRPα interaction with anti‐CD47 antibodies has been demonstrated to enable phagocytosis of AML, ALL, and solid tumor cancer cells by human macrophages in vitro.^14^, ^15^, ^16^ These findings support the premise that the observed effects of CD47 blockade in xenograft models in vivo, that is depletion of LSC from blood and bone marrow of engrafted mice and inhibition of secondary transplants, occur via macrophage‐mediated phagocytosis of CSCs.^14^, ^15^, ^16^ Additionally, anti‐CD47 antibody‐mediated phagocytosis of cancer cells has been shown to induce an antitumor T‐cell response via cross‐presentation of cancer cell antigens to the adaptive immune system,^17^ providing a second potential antitumor mechanism of action (MOA).

## 5 ANTI‐CD47 COMBINATION THERAPY MOA

The anticancer effects of the anti‐CD47 antibody in mouse models were synergistically enhanced by combining with another anticancer drug such as rituximab in a NHL model^18^ or azacytidine in an AML model,^19^ furnishing a rationale for combination clinical trials. In both cases, it was hypothesized that the observed synergy is due to augmentation of prophagocytic signals on tumor cells by the second drug as described below.

Rituximab is an anti‐CD20 monoclonal antibody that binds to normal and malignant B cells and is used to treat B‐cell lymphomas such as NHL. It is believed to act via its Fc effector functions, in part by engaging Fc receptors on NK cells and inducing antibody‐dependent cellular cytotoxicity. Macrophages also express Fc receptors and are postulated to contribute to the efficacy via antibody‐dependent cellular phagocytosis triggered by opsonization of target cells and engagement of macrophage Fc receptors by the rituximab Fc‐domain.^20^ Combining an anti‐CD47 antibody with rituximab resulted in enhanced phagocytosis of NHL cells in vitro and synergistic antitumor activity in NHL‐engrafted mice in vivo.^18^ The dual mechanisms underlying this synergy are posited to involve facilitation of macrophage phagocytosis by inhibiting the CD47 antiphagocytic signal while simultaneously stimulating phagocytosis by furnishing a strong prophagocytic signal provided by the rituximab Fc‐domain.^18^

Azacytidine is an anticancer chemotherapeutic indicated for the treatment of AML and myelodysplastic syndromes (MDS). Its mechanisms of action include inhibition of DNA methylation and cytotoxicity due to incorporation into DNA and RNA.^21^ Evidence suggests that CD47 blockade alone is insufficient to induce macrophage phagocytosis and that target cells must also express a strong prophagocytic signal in order to trigger phagocytosis.^22^ Azacytidine has been shown to induce upregulation of calreticulin^23^ which has been identified as a dominant prophagocytic signal expressed on many human cancers.^22^ In preclinical studies using an AML model, combining azacytidine with CD47 blockade resulted in enhanced macrophage‐mediated phagocytosis in vitro and enhanced antitumor activity in vivo.^19^ Analogous to the anti‐CD47‐rituximab combination, the anti‐CD47‐azacytidine synergy is postulated to be due to the dual mechanisms of blocking of the CD47 antiphagocytic signal while enhancing phagocytosis via upregulation of a prophagocytic signal, in this case, calreticulin.

## 6 CD47 BLOCKADE IN THE CLINIC

A humanized anti‐CD47 antibody (magrolimab, formerly known as Hu5F9‐G4 or 5F9) has been tested in early clinical trials. Despite widespread expression of CD47 on normal cells, CD47 blockade selectively targets cancer cells and not normal cells (except for aging red blood cells), presumably because cancer cells express prophagocytic signals that are absent from normal cells.^24^ In agreement with preclinical findings, magrolimab has been well tolerated in humans.^25^, ^26^

The efficacy outcomes of four phase 1 trials in AML, NHL, and solid tumors, using magrolimab either as monotherapy or in combination with rituximab or azacytidine, are summarized in Table 1. In three different trials with varying patient populations, magrolimab induced primarily stable disease (SD) when used as monotherapy (SD 56% in R/R AML, 46% in solid tumors, and 70% in AML/MDS with overall response rate (ORR) 0%, 6%, and 10%, respectively) (Table 1).

**TABLE 1:** Summary of clinical responses from clinical trials with magrolimab

	Phase 1	Phase 1^a^	Phase 1b^a^			Phase 1b
Patient population	R/R AML	Advanced solid tumors	R/R AML/MDS	AML	MDS	Non‐Hodgkin's lymphoma
Treatment	Magrolimab monotherapy	Magrolimab monotherapy	Magrolimab monotherapy	Magrolimab + azacytidine	Magrolimab + azacytidine	Magrolimab + rituximab
Best overall response	All phase 1 patients (n = 18)	All patients treated at ≥recommended dose (n = 35)	n = 10	n = 16	n = 13	All patients (n = 22)
OR	0 (0%)	2 (6%)	1 (10%)	11 (69%)	13 (100%)	11 (50%)
CR/CRi	0 (0%)	0 (0%)	0 (0%)	8 (50%)	7 (54%)	8 (36%)
PR	0 (0%)	2 (6%)	0 (0%)	2 (13%)	0 (0%)	3 (14%)
MLFS or marrow CR	0 (0%)	n/a	1 (10%)	1 (6%)	5 (39%)	n/a
Hematologic improvement	n/a	n/a	n/a	n/a	1 (7%)	n/a
SD	10 (56%)	16 (46%)	7 (70%)	5 (31%)	0 (0%)	3 (14%)
PD	4 (22%)	16 (46%)	2 (20%)	0 (0%)	0 (0%)	8 (36%)
Reference	26	25	27			28

*Note:* Compiled clinical responses from four clinical trials.

Abbreviations: AML, acute myelogenous leukemia; CR, complete response; CRi, complete remission with incomplete hematologic recovery; MDS, myelodysplastic syndromes; MLFS, morphologic leukemia free state; OR, overall response; PD, progressive disease; PR, partial response; SD, stable disease.

a Trials supported by CIRM.

The ORR was significantly greater when magrolimab was combined with another cancer drug as predicted by preclinical experiments, either azacytidine in AML and MDS patients or rituximab in NHL patients, over and above expected outcomes from treatment with azacytidine or rituximab alone (Table 1). In the phase 1b AML/MDS study in which magrolimab was combined with azacytidine, the ORR of the combination (100% in untreated MDS patients and 69% in untreated AML patients) was greater than expected with azacytidine alone and the median time to response (1.9 months) was more rapid than is seen with azacytidine alone.^27^ Furthermore, since magrolimab has been shown to target LSC in preclinical studies, the CD34+CD38− putative LSC frequency in the bone marrow of magrolimab + azacytidine treated AML/MDS patients was measured by flow cytometry. Analysis of bone marrow LSC frequency in study patients indicated that the combination therapy significantly decreased or eradicated LSC in responding patients. In data available for analysis, phenotypic LSC were eliminated in 63% of MDS/AML patients who had a clinical response. These early results are suggestive of successful targeting of the LSC population and consistent with this premise, no responding AML/MDS patients had as yet progressed on magrolimab + azacytidine therapy (longest responding patients in complete response [CR], 9 months and ongoing).

In the phase 1b NHL trial in which magrolimab was combined with rituximab, 95% of patients had rituximab‐refractory disease, making it unlikely that the observed response rates (50% OR with 36% CR) were due to rituximab alone and suggesting that addition of magrolimab can overcome rituximab resistance.^28^ The antitumor synergy seen with magrolimab + rituximab was attributed to activation of macrophage phagocytosis by the combined effects of augmentation of the prophagocytic signal by the rituximab Fc‐domain and inhibition of the CD47 antiphagocytic signal.^28^

Viewed from the perspective of the CSC model, patients in this study, having relapsed after a median of four previous therapies, likely had tumors that were enriched for LSC. While the underlying mechanisms of resistance to rituximab are not well understood,^29^ it is plausible to hypothesize that LSC may be refractory to rituximab and that LSC enrichment could be one mechanism of acquired resistance to rituximab. Magrolimab‐enabled macrophage‐mediated phagocytosis of the LSC population might therefore have contributed to the observed responses. It is notable that 91% of the responses were ongoing at 6 to 8 months of follow‐up, consistent with an effect on the LSC.

## 7 ROR1 AS A CSC TARGET

ROR1 is an embryonic tyrosine kinase‐like orphan receptor expressed on chronic lymphocytic leukemia (CLL) cells but not on normal B cells or most other adult cells.^30^ High‐level expression of ROR1 is an adverse prognostic marker in CLL and other cancers and is associated with accelerated disease progression and shorter OS.^31^, ^32^, ^33^ In ovarian cancer, ROR1 is highly expressed on a subpopulation of tumor cells with features of CSC and ovarian cancers with high levels of ROR1 exhibit stem cell‐like gene expression signatures.^33^ Breast cancer tumors are enriched in ROR1 positive cells following chemotherapy and show increased features of stemness.^34^ Collectively, these findings point to an association of ROR1 expression with CSCs in multiple cancers. Direct evidence was provided by experiments showing that targeting ROR1 by short hairpin RNA silencing or with an anti‐ROR1 antibody inhibited the capacity of CSCs to form colonies in vitro or to form tumors in immunodeficient mice.^33^, ^35^

## 8 ANTI‐ROR1 ANTIBODY (CIRMTUZUMAB) MOA

ROR1 is a receptor for Wnt5a and ROR1‐dependent Wnt5a signaling has been implicated in CSC maintenance and self‐renewal and also in metastasis.^36^ A number of ROR1‐dependent, Wnt5a‐mediated signaling pathways have been uncovered in CLL cells, including activation of Rac1/2, which is important for CLL cell proliferation.^36^, ^37^

A humanized anti‐ROR1 antibody (cirmtuzumab) was developed that inhibits ROR1‐dependent Wnt5a signaling by binding with high affinity to an epitope in the extracellular domain of ROR1.

Cirmtuzumab blocked Wnt5a‐enhanced proliferation of CLL cells and inhibited leukemic engraftment in a mouse model.^35^ In an ovarian cancer study, cirmtuzumab inhibited the ability of ovarian cancer CSCs to migrate, form spheroids, or engraft and form tumors in immunodeficient mice.^33^ These studies validated inhibition of ROR1 signaling by cirmtuzumab as a strategy for targeting CSCs.

## 9 ANTI‐ROR1/CIRMTUZUMAB IN THE CLINIC

Efficacy outcomes from two clinical trials using cirmtuzumab are summarized in Table 2. Cirmtuzumab as monotherapy was assessed in a phase 1 dose‐finding trial in CLL patients with relapsed/refractory disease.^38^ Patients received four biweekly infusions with doses ranging from 15 μg/kg to 20 mg/kg. ROR1 is not expressed on normal adult cells and consistent with this expression pattern, cirmtuzumab was well tolerated with no dose‐limiting toxicities. As shown in Table 2, while no patients met criteria for objective response, 77% of patients had SD upon completing treatment despite having objective signs of disease progression upon enrolling in the study. Viewed from the perspective of the CSC model, a possible interpretation of this result is that inhibition of ROR1‐signaling by cirmtuzumab, which was confirmed by analysis of patient samples from the study, inhibited the CSC population driving tumor progression. A transcriptome analysis of CLL cells from study patients supports this thinking. Analysis of post‐treatment samples revealed a highly significant reversal of a stemness gene expression signature that was observed in pretreatment CLL cells.^38^

**TABLE 2:** Summary of clinical responses from clinical trials with cirmtuzumab

	Phase 1^a^	Phase 1/2^a^ (ongoing)
Patient population	R/R CLL	CLL
Treatment	Cirmtuzumab monotherapy	Cirmtuzumab + ibrutinib
Best overall response	Evaluable patients n = 22	Interim data n = 12
OR	0 (0%)	11 (91.7%)
CR/CRi	0 (0%)	3 (25%)
PR/PR‐L	0 (0%)	8 (66.7%)
SD	17 (77%)	1 (8.3%)
PD	5 (23%)	0 (0%)
Reference	38	39

Note: Compiled clinical responses from two clinical trials.

Abbreviations: CLL, chronic lymphocytic leukemia; CR, complete response; CRi, CR with incomplete marrow recovery; OR, overall response; PD, progressive disease; PR, partial response; PR‐L, PR with lymphocytosis; SD, stable disease.

a Trials supported by CIRM.

Ibrutinib, a Bruton's tyrosine kinase (BTK) inhibitor that blocks B‐cell receptor signaling, is an effective therapy for patients with CLL although it does not completely eradicate disease.^40^ In preclinical experiments, cirmtuzumab and ibrutinib were shown to target independent signaling pathways and in human CLL xenografts, combined treatment with both cirmtuzumab and ibrutinib was more effective than either drug alone in reducing the number of CLL cells.^41^

These preclinical experiments provided a rationale for combining cirmtuzumab with ibrutinib clinically and a phase 1/2 trial is currently testing the combination in CLL patients. Interim data from the first 12 patients in the phase 1 portion of the trial showed an overall objective response rate of 91.7% with a CR rate of 25% after 24 to 48 weeks of treatment^39^ (Table 2). These early results, in particular the encouraging CR rate, which is higher than would be expected with ibrutinib alone, are suggestive of synergy between the two drugs as predicted by the preclinical data.

## 10 DISCUSSION

While acknowledging the extreme heterogeneity and complexity of human tumors, a simplified interpretation of the CSC model predicts the following: Therapies that target bulk tumor but not the CSC subpopulation may cause tumor shrinkage, inducing objective responses and even complete responses, but the responses will not be durable. In contrast, a therapy that targets primarily the CSC subpopulation but not bulk tumor cells might arrest tumor progression without inducing tumor shrinkage, resulting in SD without objective responses. A key prediction of the CSC model is that durable objective/complete responses will only be achieved by combined targeting of both bulk tumor cells and the CSC subpopulation.

The two CSC‐targeted therapeutic strategies described above work by completely different mechanisms. Yet it is interesting to note a number of parallels. First, in both cases, high‐level expression of the target in human tumors is a negative prognostic marker, predicting shorter OS. Second, clinical evaluation of these strategies as monotherapy resulted primarily in SD, a result consistent with the predictions of the CSC model. Third, there is strong preclinical rationale for combination therapy with other anticancer drugs and although it is still too early to tell what the clinical combination trials will show, initial results appear promising and are consistent with additive or synergistic effects. Importantly, both approaches are well tolerated in humans and there appear to be no limiting toxicities.

While overall response rate (ORR) is an accepted endpoint for evaluating efficacy of anticancer therapies, it does not test the basic premise of the CSC model. More meaningful endpoints will be duration of response, progression‐free survival, relapse‐free survival, and ultimately, OS. It remains to be seen whether the approaches described in this article will lead to long‐term, durable responses when combined with another anticancer therapy, as predicted by the CSC model.

## CONFLICT OF INTEREST

The author declared no potential conflicts of interest.
